# Differential Effect of Viable Versus Necrotic Neutrophils on *Mycobacterium tuberculosis* Growth and Cytokine Induction in Whole Blood

**DOI:** 10.3389/fimmu.2018.00903

**Published:** 2018-04-27

**Authors:** David M. Lowe, Julie Demaret, Nonzwakazi Bangani, Justine K. Nakiwala, Rene Goliath, Katalin A. Wilkinson, Robert J. Wilkinson, Adrian R. Martineau

**Affiliations:** ^1^Wellcome Centre for Infectious Diseases Research in Africa, Department of Medicine, Institute of Infectious Diseases and Molecular Medicine, University of Cape Town, Cape Town, South Africa; ^2^Department of Medicine, Imperial College London, London, United Kingdom; ^3^Institute of Immunity and Transplantation, University College London, London, United Kingdom; ^4^Barts and The London School of Medicine, Blizard Institute, Queen Mary University of London, London, United Kingdom; ^5^Department of Biomedical Sciences, Institute of Tropical Medicine, Antwerp, Belgium; ^6^Department of Biomedical Sciences, University of Antwerp, Antwerp, Belgium; ^7^The Francis Crick Institute, London, United Kingdom

**Keywords:** neutrophil, mycobacteria, tuberculosis, necrosis, viability

## Abstract

Neutrophils exert both positive and negative influences on the host response to tuberculosis, but the mechanisms by which these differential effects are mediated are unknown. We studied the impact of live and dead neutrophils on the control of *Mycobacterium tuberculosis* using a whole blood bioluminescence-based assay, and assayed supernatant cytokine concentrations using Luminex™ technology and ELISA. CD15+ granulocyte depletion from blood prior to infection with *M. tuberculosis*-lux impaired control of mycobacteria by 96 h, with a greater effect than depletion of CD4+, CD8+, or CD14+ cells (*p* < 0.001). Augmentation of blood with viable granulocytes significantly improved control of mycobacteria by 96 h (*p* = 0.001), but augmentation with necrotic granulocytes had the opposite effect (*p* = 0.01). Both augmentations decreased supernatant concentrations of tumor necrosis factor and interleukin (IL)-12 p40/p70, but necrotic granulocyte augmentation also increased concentrations of IL-10, G-CSF, GM-CSF, and CCL2. Necrotic neutrophil augmentation reduced phagocytosis of FITC-labeled *M. bovis* BCG by all phagocytes, whereas viable neutrophil augmentation specifically reduced early uptake by CD14+ cells. The immunosuppressive effect of dead neutrophils required necrotic debris rather than supernatant. We conclude that viable neutrophils enhance control of *M. tuberculosis* in blood, but necrotic neutrophils have the opposite effect—the latter associated with induction of IL-10, growth factors, and chemoattractants. Our findings suggest a mechanism by which necrotic neutrophils may exert detrimental effects on the host response in active tuberculosis.

## Introduction

Neutrophils interact with *Mycobacterium tuberculosis* (1, 2) and are important in the host immune response to this pathogen (3). However, their influence on the outcome of infection remains controversial: while they may have a protective role in early infection (3), evidence exists for a detrimental effect in established tuberculosis disease (4, 5).

Literature indicates that neutrophils have a profound impact on the development of innate and acquired immune responses (6, 7), but this has not been significantly studied in tuberculosis. *In vivo* data have revealed a lower percentage of interferon (IFN)-γ expressing CD4+ T cells in bronchoalveolar lavage samples from cavities, which are rich in neutrophils (8), but a causative relationship has not been established.

Neutrophils are also frequently demonstrated to associate with tuberculosis immunopathology in animals (9, 10) and this appears to be specifically associated with necrosis (11). We have also previously observed that neutrophil antimycobacterial activity is impaired during HIV-1 infection (12) and that this is associated with rapid neutrophil necrosis. Others have also noted a neutrophilic response with significant necrosis in the tuberculous lesions of HIV-1 co-infected persons, in association with impaired tumor necrosis factor (TNF) staining (13). Animal models link neutrophil necrosis with pathogen virulence and host susceptibility (14), or link overall necrosis of granulomas with bacillary burden (15), suggesting that this pattern of cell death may correlate with severity of disease. More recently, it has been demonstrated *via* immunohistochemistry that neutrophil markers in human tuberculous granulomas associate with areas of necrosis and with interleukin (IL)-10, while a pronounced neutrophilic necrosis response correlates with enhanced numbers of *M. tuberculosis* organisms (16).

We therefore investigated the influence of viable and necrotic neutrophils on the immune response to tuberculosis. We first examined the cytokine and chemokine profile of supernatants aspirated from whole blood which had been depleted of neutrophils or various other cell types before incubation with *M. tuberculosis*. We also examined the impact of augmenting blood with additional autologous neutrophils [as they are recruited in large numbers to sites of tuberculosis infection (1)] on both the control of infection and the cytokine response, using an *ex vivo* whole blood model (17, 18). We then investigated whether the effect of necrotic neutrophils contrasted with viable neutrophils in modulating the control of and response to infection. These experiments modeled a situation of neutrophilia with rapid cell death, as may be seen in necrotic granulomata, in tuberculosis-HIV co-infection or in severe tuberculosis disease.

## Materials and Methods

### Patients and Recruitment

Participants for depletion experiments were recruited among people who had recently tested negative for HIV in Khayelitsha, South Africa, either at the Ubuntu Site B clinic or the Khayelitsha Youth Centre. Augmentation and phagocytosis experiments were performed using blood from healthy lab donors.

### Ethics

The studies were approved by the University of Cape Town Research Ethics Committee (HREC 545/2010) in South Africa and NHS Research Ethics Committee (REC 08-H0720-46) in the UK and performed in accordance with the Declaration of Helsinki. Participants provided written informed consent.

### Organisms and Labeling

The development of luminescent mycobacteria, including plasmid construction and electroporation, has been described previously (19). 1.5 ml vials of *M. tuberculosis*-lux stored at −80°C were thawed and added to 15 ml 7H9 (Becton Dickinson)/ADC (Becton Dickinson) growth medium containing 0.05% Tween 80 (Sigma) and 1 mcl/ml hygromycin B (Roche diagnostics). Organisms were cultured to mid-log phase (72 h) before use. Luminescence was measured as previously described (2). *M. bovis* BCG-lux organisms were processed similarly but cultured in 20 ml 7H9/ADC. Non-luminescent *M. bovis* BCG were cultured to mid-log phase in 7H9/ADC (monitored by optical density) and labeled with FITC when required for experiments, as previously described (2).

### Cell Depletion and Neutrophil Isolation

For comparative depletion experiments, heparinized blood was divided into aliquots and incubated with Miltenyi MicroBeads at 2–8°C. Volumes and incubation times were optimized for each antigen and achieved approximately 90% depletion of the target cell [anti-CD4: 100 mcl/ml, 30 min; anti-CD8: 50 mcl/ml, 15 min; anti-CD14: 100 mcl/ml, 30 min; anti-CD15: 50 mcl/ml, 15 min; Basic (unconjugated) MicroBeads used for controls: 50 mcl/ml, 15–30 min; see Figure S1 in Supplementary Material]. Blood was diluted 1:1 with RPMI-1640 and passed through Miltenyi Biotec LS columns supported in magnets (MidiMACS Separation Unit, Miltenyi Biotec); columns had been pre-“primed” with 3 ml MACS buffer [0.5% bovine serum albumin (Sigma) and 2 mM EDTA (Sigma)], all as previously described (2). Depleted blood was collected in Universal containers. Granulocyte isolation using discontinuous Percoll gradient was performed as previously described (2). Neutrophils were rendered necrotic (when required) *via* heat shock at 60°C for approximately 20 min, until all cells were trypan blue (Sigma) positive by microscopy, and then allowed to cool.

### Whole Blood Lux Assay

This was performed as previously described in detail (17, 18). Briefly, for comparative depletion experiments, approximately 5 × 10^5^ cfu mid-log phase *M. tuberculosis*-lux in 100 mcl PBS was added to triplicate samples of 900 mcl specific cell-depleted or undepleted (Basic MicroBead-treated) blood, already diluted 1:1 with RPMI as described above. For augmentation experiments, the same inoculum was added to triplicate samples of 450 mcl venous blood pre-mixed 1:1 with either RPMI-1640, viable neutrophils in RPMI-1640 or necrotic neutrophils in RPMI-1640. For each donor, inocula were identical between different depletion/augmentation conditions. Samples were incubated at 37°C on a rocking platform for 96 h; mycobacterial luminescence was measured after removal of supernatants, cell lysis with 10 ml water, centrifugation at 2,000 × *g* for 10 min and resuspension in 1 ml PBS. Supernatants did not contain significant quantities of mycobacteria despite effective release from cells during lysis (Figure S2 in Supplementary Material). Uninfected samples served as controls for cytokine analysis. For comparison of the effects of necrotic neutrophil debris versus supernatants, samples were processed identically except that all Percoll-isolated neutrophils were heat-shocked and the necrotic neutrophils were then centrifuged at 1,200 × *g* for 6 min. Supernatants were removed and the necrotic cell pellet resuspended; 450 mcl aliquots of reserved whole blood were then mixed 1:1 with necrotic neutrophils, necrotic neutrophil supernatant, or RPMI-1640 alone in duplicate (plus one uninfected sample per donor and condition). The infecting organism in these experiments was *M. bovis* BCG, added at the same multiplicity of infection.

### Phagocytosis Assay

Samples were prepared as for the lux assay, with the addition of viable, necrotic, or no autologous neutrophils to venous blood, in duplicate per experimental condition. Samples were infected with 100 mcl FITC-labeled BCG organisms diluted according to optical density to approximately 2 × 10^6^ CFU/ml. For each donor, inocula were identical between different augmentation conditions. Samples were mixed and incubated at 37°C for 1 h.

Subsequently, two 200 mcl aliquots were taken from the samples, to which 10 mcl anti-CD14-PE (Becton Dickinson) and 1 mcl BD Horizon™ Fixable Viability Stain 450 (Becton Dickinson) were added. After 20 min, red blood cells were lysed *via* 15-min incubation with 2 ml 1× BD Pharm Lyse™ (Becton Dickinson). Samples were then centrifuged at 600 × *g* for 5 min, resuspended in 1 ml Stain Buffer (BD Pharmingen™) with 12.5 mcl filtered trypan blue (Sigma), centrifuged again, fixed in 500 mcl Cytofix (Becton Dickinson) for 15 min and finally resuspended in 1 ml Stain Buffer. Samples were processed on a BD Fortessa flow cytometer and data analyzed with FlowJo software (Treestar). Figure S3 in Supplementary Material details the gating strategy employed; results are presented as mean of duplicate results per donor/condition.

### TNF and IL-10 ELISA

These were performed on supernatants according to manufacturer’s instructions (Peprotech, Rocky Hill, NJ, USA). Final dilutions were fivefold; results are presented as mean of duplicate samples per patient/condition. Undetectable readings were assigned an arbitrary value of 1 pg/ml (below the limit of detection of the assay).

### Quantiferon-TB Gold™ In-Tube IFN γ Release Assay

This was performed according to manufacturer’s instructions.

### Luminex™

Supernatants from 96-h blood samples infected with *M. tuberculosis*-lux were pooled across triplicates per participant/condition, filtered twice through 0.22 µm filter plates (Merck Millipore, Billerica, MA, USA) with centrifugation at 3,000 × *g* and diluted appropriately according to preliminary results: final dilutions were either 4-fold or 10-fold depending on the experiment. The Invitrogen 30-plex kit (Invitrogen, Carlsbad, CA, USA) was used according to manufacturer’s instructions, but we used a lower volume of beads: either 12.5 or 8.4 mcl. Plates were read on a calibrated Luminex 200™ reader and results calculated by the Luminex 200™ software.

### Statistics

Parametric data were analyzed using Student’s *t*-tests when comparing two groups (paired when appropriate) or one-way ANOVA for comparing three or more groups with *post hoc* correction. Non-parametric data were analyzed using Mann–Whitney *U*-tests or Wilcoxon signed rank tests for two groups and Kruskal–Wallis or Friedman testing with *post hoc* Dunn’s correction for three or more groups.

All statistical analyses were performed using SPSS Version 18.0 (SPSS Inc., Chicago, IL, USA) or GraphPad Prism version 4.0 or later. All reported *p*-values are two-sided; *p* < 0.05 was inferred as significant.

### Principal Component Analysis (PCA)

Principal component analysis and associated three-dimensional plots were generated using Qlucore™ Omics Explorer v2.3 software. Multi-group comparison was used to identify Luminex™-measured cytokines or chemokines that were differentially abundant between the supernatants of three conditions (blood augmented with viable neutrophils, necrotic neutrophils, or medium only). A *p*-value of maximum 0.05 and *q*-value of maximum 0.1 were used as thresholds. Individual points in the plots represented one donor in one augmentation condition and their position in the plot was determined by the combined effects of all parameters measured for the sample that significantly contributed to the overall between-group difference. The distance between sample points represents Euclidean distance and was calculated using all parameters that significantly contribute to the overall between-group difference. Component vectors are displayed, along with a percentage figure signifying the proportion of the variability in the data that each component accounts for.

## Results

### Depletion of Neutrophils From Whole Blood Impairs Mycobacterial Control More Than Depletion of Other Cell Types

**Figure 1:**
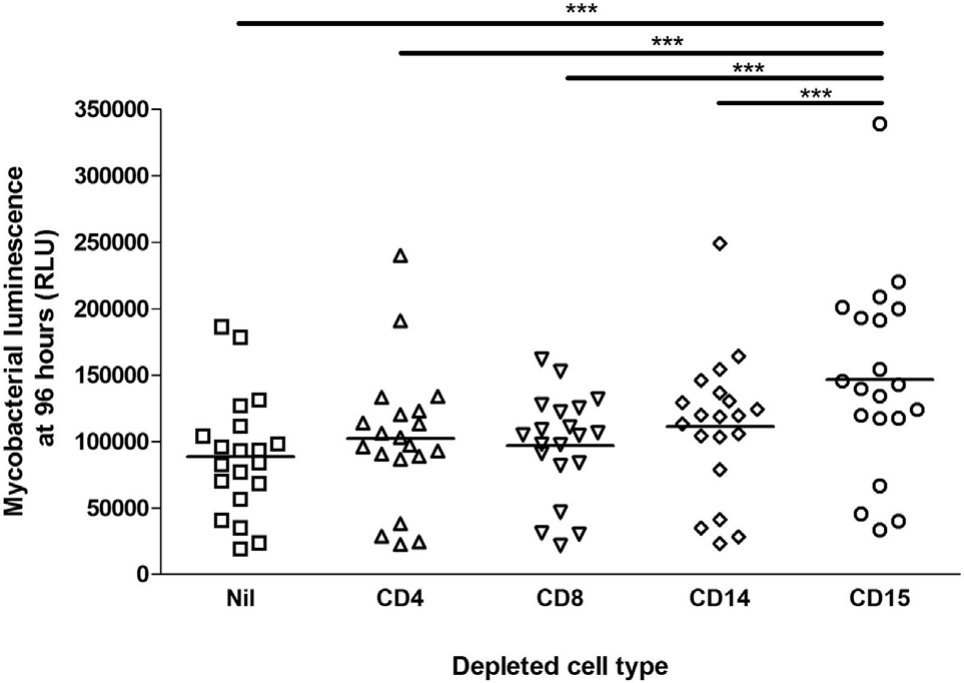
Impact of cell depletions on control of mycobacterial luminescence in whole blood. Blood was taken from 20 donors. 450 mcl of blood depleted of CD4+, CD8+, CD14+, CD15+ or no cells (in triplicate per donor per condition), diluted 1:1 with RPMI-1640, was infected with approximately 5 × 10^5^ CFU *Mycobacterium tuberculosis*-lux in 100 mcl PBS. Samples were incubated on a rocking plate (20 rpm) at 37°C for 96 h before removal of supernatants, lysis of red blood cells with water, resuspension in 1 ml PBS and measurement of mycobacterial luminescence on at least two 100 mcl aliquots. Results presented are the mean of all measurements across triplicate samples for the relevant condition. ***p < 0.001 (one-way ANOVA).

Aliquots of blood from 20 healthy, HIV-uninfected persons (10 with positive results from Quantiferon-TB Gold In-Tube IFN-γ release assay) were depleted of CD4+, CD8+, CD14+, or CD15+ cells *via* Miltenyi MACS magnetic beads or were incubated with non-conjugated beads as controls. This blood was then infected with *M. tuberculosis*-lux and incubated in rocking tubes for 96 h, as per the previously described whole blood lux assay (17). The mean (SD) neutrophil count in mock-depleted blood [treated with Basic (unconjugated) Microbeads, diluted 1:1 with RPMI-1640] was 1.42 ± 0.53 × 10^9^/l and in CD15-depleted blood was 0.20 ± 0.17 × 10^9^/l. Figure 1 details the impact of cell depletion on the 96-h luminescence of *M. tuberculosis-*lux. CD15+ cell (granulocyte) depletion had a greater effect than depletion of any other cell type on mycobacterial luminescence (a surrogate of mycobacterial metabolism and/or number) at 96 h (mean RLU per 100 mcl 88,686 ± 44,734 in mock-depleted blood versus 146,681 ± 72,663 in CD15-depleted blood, 102,262 ± 52,568 in CD4-depleted blood, 97075 ± 38993 in CD8-depleted blood and 111,338 ± 52,879 in CD14-depleted blood; *p* < 0.001 for CD15-depleted versus any other condition). We note that by pairwise comparison versus undepleted blood, both CD4 depletion (*p* = 0.016) and CD14 depletion (*p* = 0.004) also impaired control of mycobacterial luminescence, although the effects were more modest than with CD15 depletion.

### CD15+ Cell Depletion Has Relatively Little Effect on the Cytokine and Chemokine Concentrations in Supernatants of *M. tuberculosis*-Infected Blood

**Figure 2:**
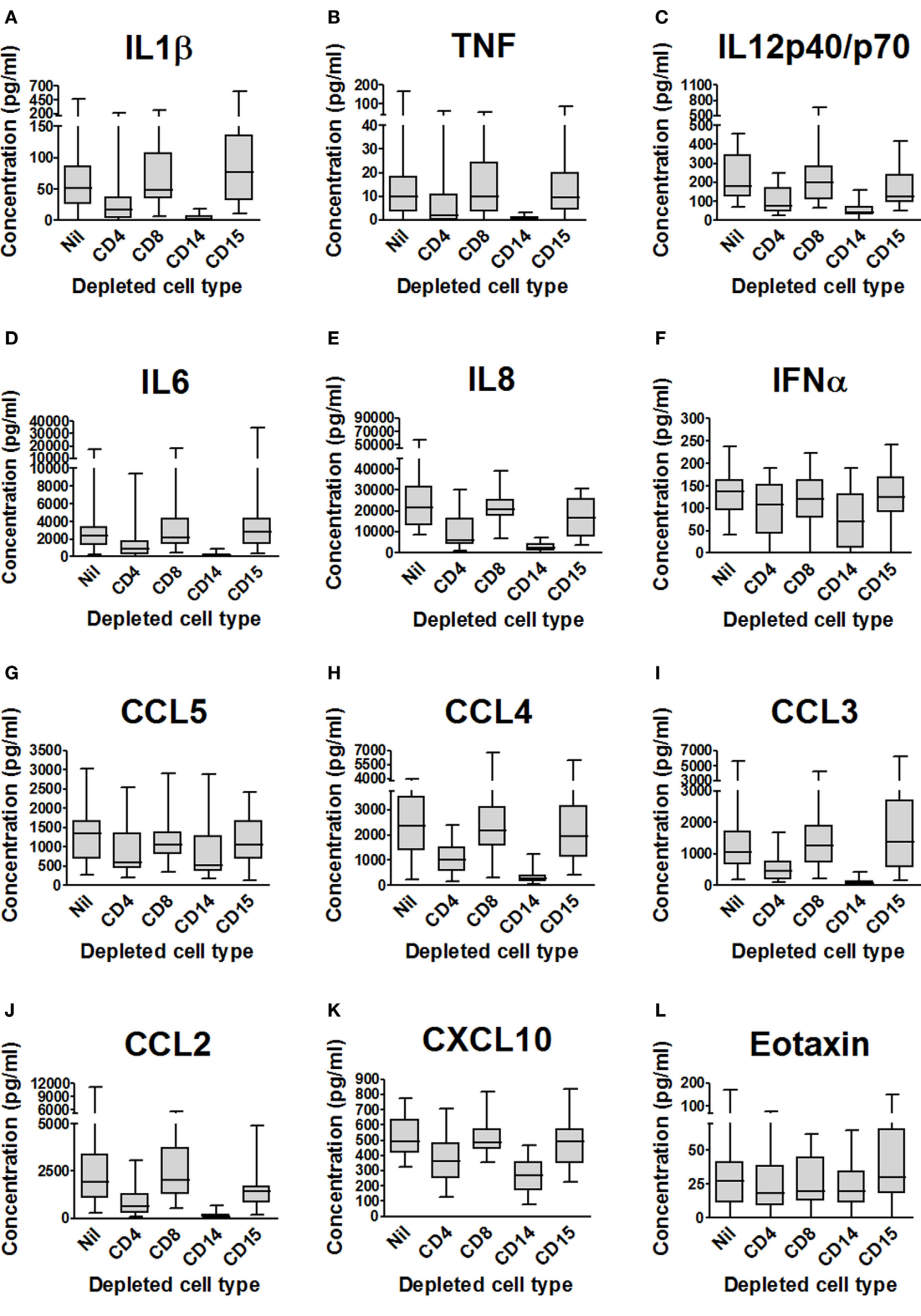

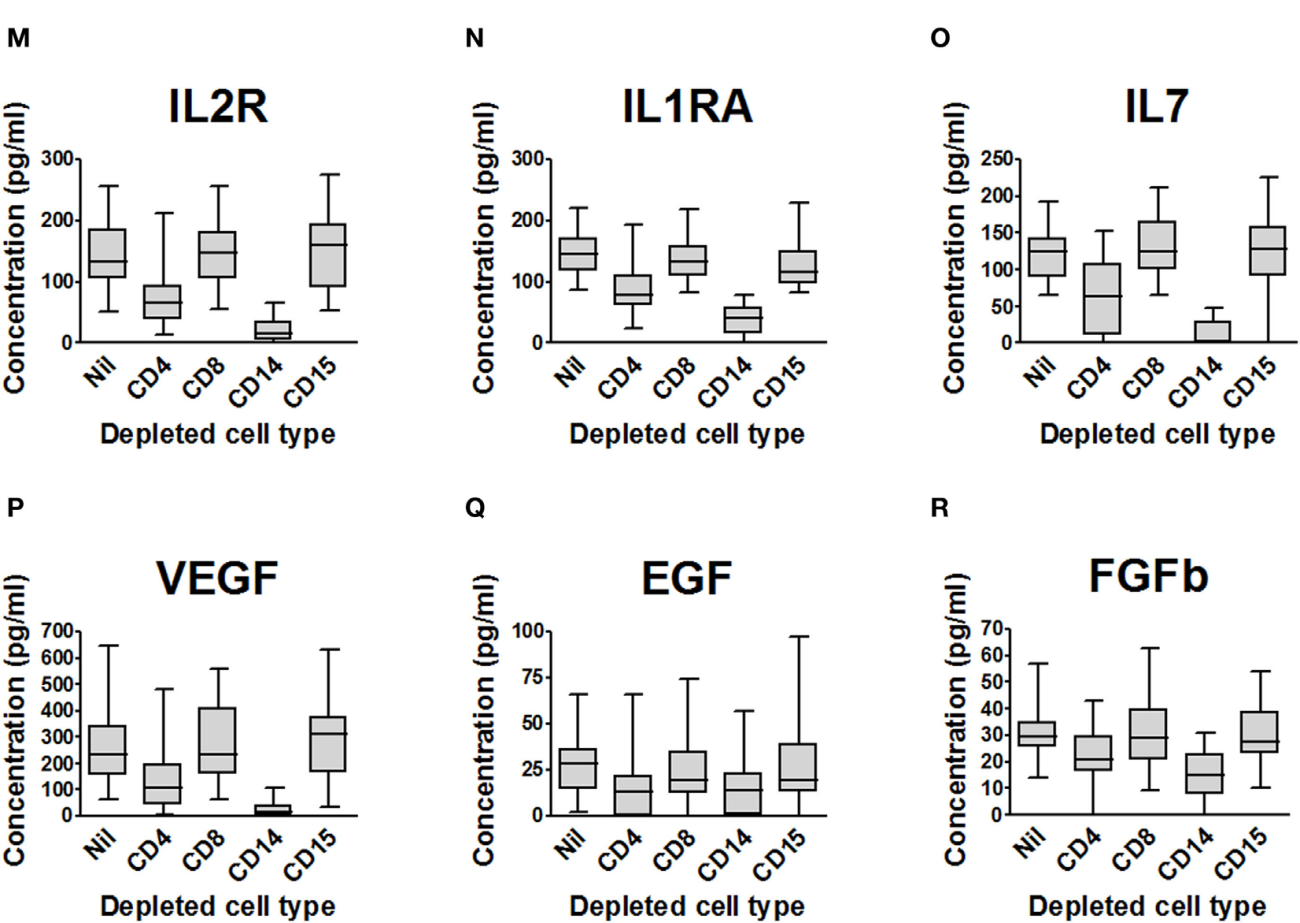
Impact of cell depletions on supernatant cytokines and chemokines from *Mycobacterium tuberculosis*-infected blood. **(A–R)**. Supernatants from the experiments presented in Figure 1 (blood from 20 donors depleted of CD4+, CD8+, CD14+, CD15+ or no cells and infected with *M. tuberculosis*-lux for 96 h) were aspirated and stored at −80°C until analysis by Luminex™ technology. Box and whisker plots represent median, interquartile range, minimum and maximum values for each analyte. Abbreviations: VEGF, vascular endothelial growth factor; TNF, tumor necrosis factor; IFN, interferon; IL, interleukin; IL-2R, interleukin-2 receptor; IL-1RA, interleukin-1 receptor antagonist; FGFb, fibroblast growth factor basic; EGF, epidermal growth factor; CXCL, C-X-C motif ligand; CCL, C-C chemokine ligand; CD, cluster of differentiation.

Luminex™ analyses of cytokines and chemokines in the supernatants from depletion experiments (see above) are summarized in Figure 2 and Table 1, which indicate those analytes significantly affected by cell depletion after correction for multiple pairwise comparisons by the Bonferroni methodology. Uninfected control samples showed low or undetectable levels of most analytes, although when they were detected there was evidence that cell depletion by itself had a significant effect on cytokine and chemokine responses; subtracting baseline values from uninfected controls thus removes some of the biological effect of the depletion and we, therefore, present analysis on raw data.

**Table 1:** Summary of impact of cell depletions on supernatant cytokines and chemokines from *Mycobacterium tuberculosis*-infected blood.

	Depleted cell			
	CD4	CD8	CD14	CD15
VEGF	↓		↓	
TNF	↓		↓	
IFN-α	↓		↓	
IL-8	↓		↓	
IL-7	↓		↓	
IL-6	↓		↓	
IL-2R	↓		↓	
IL-1RA	↓		↓	
IL-1β	↓		↓	
IL-12p40p70	↓		↓	
FGFb	↓		↓	
Eotaxin				
EQF			↓	
CXCL10	↓		↓	
CCL5	↓			
CCL4	↓		↓	
CCL3	↓		↓	
CCL2	↓		↓	↓

*Down arrows indicate those analytes which remain significantly decreased in the depleted condition compared to mock-depleted (*p* < 0.05, Wilcoxon matched pairs) after adjustment for multiple comparisons *via* the Bonferroni methodology*.

*VEGF, vascular endothelial growth factor; TNF, tumor necrosis factor; IFN, interferon; IL, interleukin; IL-2R, interleukin-2 receptor; IL-1RA, interleukin-1 receptor antagonist; FGFb, fibroblast growth factor basic; EGF, epidermal growth factor; CXCL, C-X-C motif ligand; CCL, C-C chemokine ligand; CD, cluster of differentiation*.

Depletion of CD8+ cells had no effect on the cytokine profile of 96-h blood supernatants, while the effect of depletion of CD4+ cells significantly reduced supernatant concentrations of 16 predominantly Th1-associated cytokines, innate inflammatory cytokines, and chemokines. CD14+ cell depletion markedly reduced the concentration of 16 similar molecules. CD15+ cell depletion had less effect on the supernatant analyte profile than CD4+ or CD14+ cell depletion, but after correction for multiple comparisons there was significantly (*p* < 0.05) reduced CCL2 concentration versus undepleted blood [median (IQR) concentration 1,420 (807–1,684) ng/ml in CD15-depleted blood supernatants versus 1,903 (1,084–3,386) ng/ml in mock-depleted blood supernatants].

### Addition of Viable Neutrophils to Human Whole Blood Improves Mycobacterial Control at 96 h, While Addition of Necrotic Neutrophils Has the Opposite Effect

We proceeded to investigate the effect of augmenting blood with either viable or necrotic neutrophils on mycobacterial restriction and cytokine release. Neutrophils from nine donors were isolated by discontinuous Percoll gradient and divided into two equal aliquots; one half was then heat-shocked at 60°C (and subsequently cooled) and either viable neutrophils, necrotic neutrophils, or medium only were added to aliquots of the same donor’s blood (kept aside at the time of venepuncture, for a maximum of 2 h). The whole blood lux assay was conducted as previously described (17, 18).

**Figure 3:**
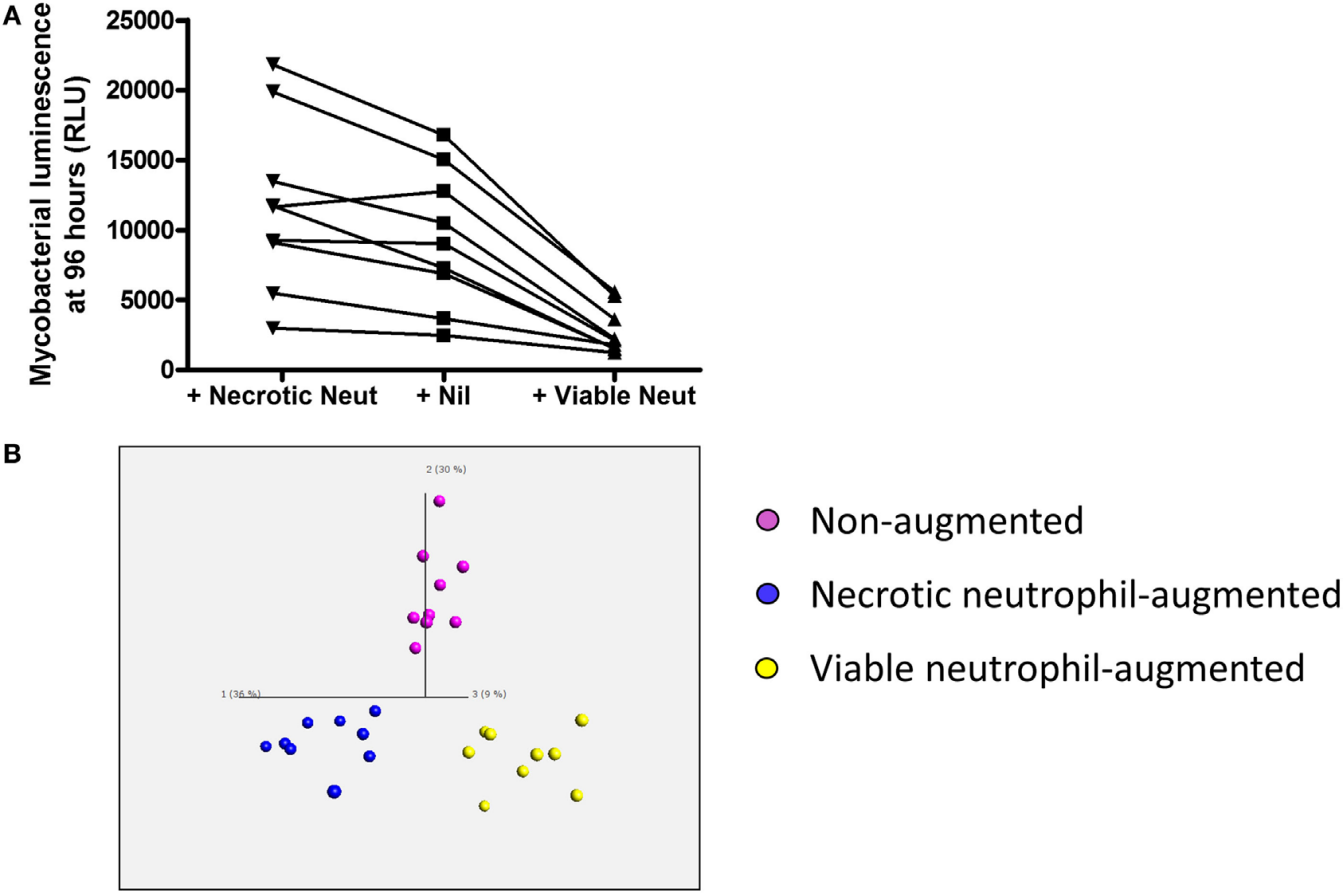
Impact of viable and necrotic neutrophil augmentation on control of mycobacterial luminescence by whole blood and on supernatant cytokines and chemokines from *Mycobacterium tuberculosis*-infected blood. **(A)** 100,000 RLU of *M. tuberculosis*-lux in 100 mcl PBS was inoculated into samples of 450 mcl whole blood plus either 450 mcl Percoll-isolated autologous neutrophils in RPMI-1640 heat-shocked at 60°C for 20 min and allowed to cool (“+Necrotic Neut”), 450 mcl RPMI-1640 only (“+Nil”), or 450 mcl room temperature Percoll-isolated autologous neutrophils in RPMI-1640 (“+Viable Neut”). After 96-h incubation, red blood cells were lysed and luminescence was measured on at least two aliquots of 100 mcl. Results are shown from nine independent donors. **(B)** Three-dimensional principal component analysis (PCA) plot generated using cytokine/chemokines that significantly contribute to differentiation between supernatants of augmentation conditions (calculated using multi-group comparison; purple = non-augmented, blue = necrotic neutrophil-augmented, yellow = viable neutrophil-augmented). PCA is a technique to reduce the dimensionality of complex datasets by transforming the data to a coordinate system. The first three coordinates (principal components) are represented as a 3D plot. The first principal component accounts for as much variability as possible within the data, and each succeeding component accounts for the next highest proportion of the variability possible, but under the constraint that it is not correlated with preceding components. This allows visualization of the differences between patient samples and analytes within complex datasets. Individual points represent one donor in one augmentation condition and their position in the plot is determined by the combined effects of all parameters measured for the sample that significantly contribute to the overall between-group difference. Component vectors for the three main components are displayed, along with a percentage figure signifying the proportion of the variability in the data that each component accounts for. Analysis is presented using raw values from infected supernatants.

Overall neutrophil purity assessed by Coulter counting was 97.79 ± 1.16%. The non-augmented mean ± SD neutrophil count was 2.10 ± 0.75 × 10^9^/l versus 9.00 ± 3.30 × 10^9^/l in those samples augmented with viable neutrophils (*p* < 0.0001). Lymphocyte counts were not significantly different: 1.18 ± 0.53 × 10^9^/l in non-augmented versus 1.30 ± 0.59 × 10^9^/l (*p* = 0.09) in neutrophil-augmented blood. Luminescence readings at 96 h post-infection (performed in at least duplicate per donor per condition) were 9,379 ± 4,888 RLU per 100 mcl for non-augmented blood. Luminescence was significantly decreased in samples augmented with viable neutrophils (2,766 ± 1,672 RLU per 100 mcl, *p* < 0.001), and 96-h luminescence correlated inversely with the number of neutrophils using all data from non-augmented or viable-augmented samples (*p* < 0.001). Conversely, augmentation with necrotic neutrophils increased mean luminescence at 96 h post-infection to 11,722 ± 6,132 RLU per 100 mcl (*p* = 0.013 versus no augmentation, Figure 3A).

### Addition of Viable or Necrotic Neutrophils to Human Whole Blood Results in a Significant Alteration of Cytokine and Chemokine Response

Supernatants from these experiments were assayed by Luminex™ 30-plex technology. In total, 26 cytokines or chemokines were detectable with a median value greater than the lower limit of detection. To identify differences in inflammatory profile between the three conditions (non-augmented, viable neutrophil-augmented, or necrotic neutrophil-augmented) we employed Qlucore™ Omics Explorer v2.3 to perform PCA. Initial exploration of data suggested that neutrophil manipulation by itself had a significant effect on cytokine and chemokine responses; subtracting uninfected control values, therefore, removes some of the biological effect and we present analysis on raw data.

**Figure 4:**
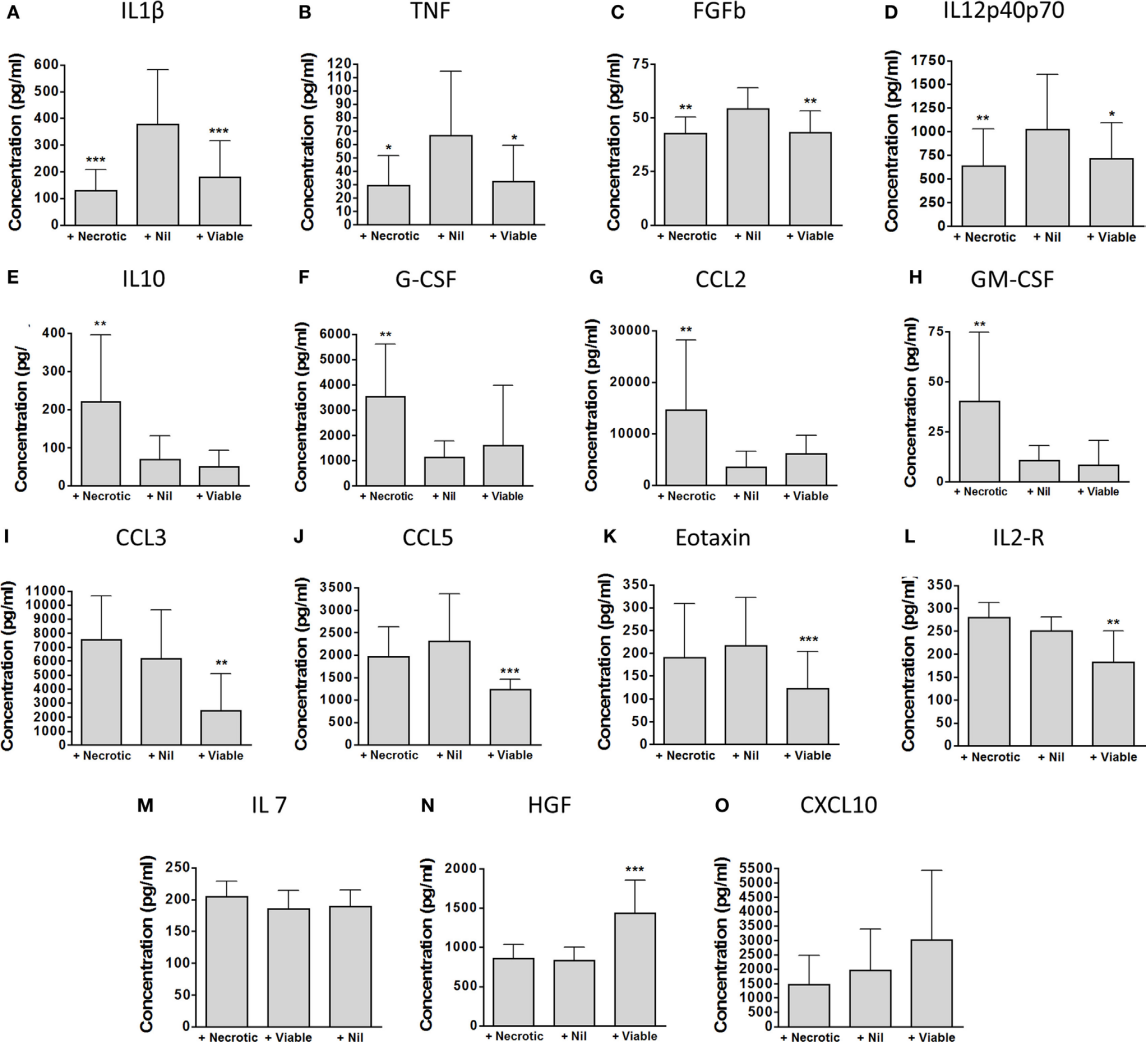
Detailed impact of viable and necrotic neutrophil augmentation on supernatant cytokines and chemokines from *Mycobacterium tuberculosis*-infected blood. **(A–O)** Cytokines and chemokines were measured in 96-h supernatants of *M. tuberculosis*-infected blood which had been augmented with necrotic autologous neutrophils (“+Necrotic”), with medium only (“+Nil”), or with viable autologous neutrophils (“+Viable”). Bars represent mean ± SD for each analyte found to be significant in principal component analysis. Presented are *p*-values from one-way ANOVA with Bonferroni correction comparing both augmented conditions with the medium-only control: *p < 0.05, **p < 0.01, ***p < 0.001. Data are from nine separate donors in four independent experiments. Abbreviations: IL, interleukin; TNF, tumor necrosis factor; FGFb, fibroblast growth factor basic; G-CSF, granulocyte-colony stimulating factor; GM-CSF, granulocyte macrophage-colony stimulating factor; CCL, C-C chemokine ligand; CXCL, C-X-C motif ligand; IL-2R, interleukin-2 receptor; HGF, hepatocyte growth factor.

Principal component analysis showed clear differences in inflammatory profiles between the non-augmented, viable neutrophil-augmented, and necrotic neutrophil-augmented conditions (Figure 3B; Table S1 in Supplementary Material details *p*- and *q*-values). Figure 4 presents analyte concentrations and displays results from one-way ANOVA for each cytokine or chemokine found to contribute to the separation of groups in PCA. Maximal cytokine responses were seen in the non-augmented blood. Viable neutrophil augmentation was broadly anti-inflammatory, reducing supernatant concentrations of IL-1β, TNF, IL-12p40/p70, CCL3, CCL5, interleukin-2 receptor (IL-2R), and fibroblast growth factor basic (FGFb); only hepatocyte growth factor (HGF) was increased. The addition of necrotic neutrophils to blood was also associated with lower supernatant concentrations of IL-1β, TNF, IL-12p40/p70, and FGFb, but simultaneously provoked an increase in IL-10, G-CSF, GM-CSF, and CCL2.

### Addition of Necrotic Neutrophils to Whole Blood Reduces Phagocytosis Overall, While Addition of Viable Neutrophils Reduces Phagocytosis of Mycobacteria Specifically by CD14+ Cells

To investigate the mechanism by which neutrophils elicited an anti-inflammatory response to mycobacterial infection, we measured phagocytosis of FITC-labeled *M. bovis* BCG by CD14+ cells (shown earlier to be the major source of cytokines and chemokines in the whole blood model, Figure 2).

**Figure 5:**
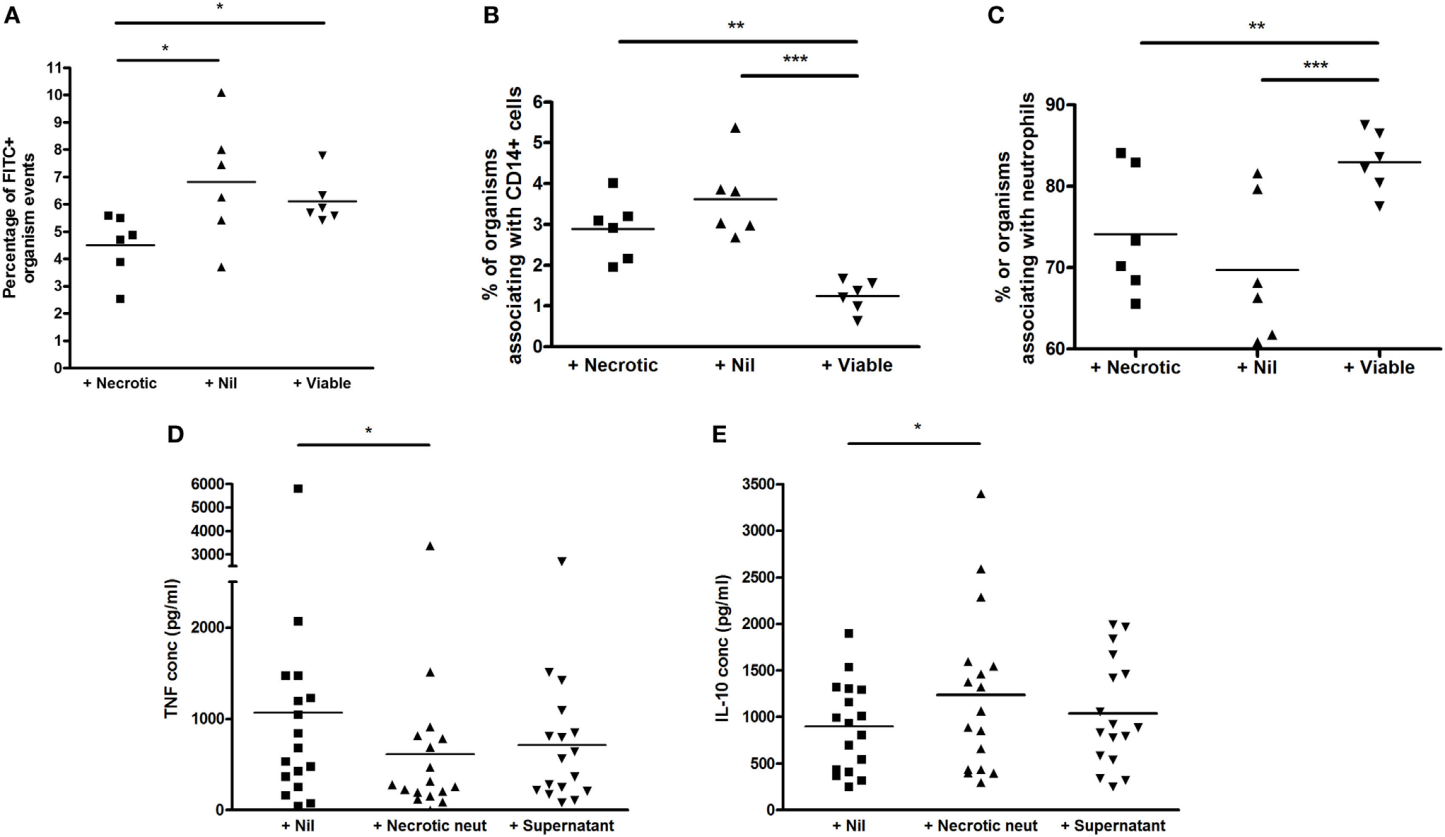
Effect of viable and necrotic neutrophil augmentation on phagocytosis of FITC-labeled *M. bovis* BCG, and differential effect of necrotic neutrophil augmentation versus supernatant of necrotic neutrophils on cytokine release in blood. **(A–C)** Blood from six donors (four independent experiments) was augmented with viable neutrophils, necrotic neutrophils, or medium alone, infected with 2 × 10^5^ CFU FITC-labeled *M. bovis* BCG and incubated for 1 h at 37°C. 2 × 100 mcl aliquots were taken from each sample, incubated with CD14-PE and Viability Dye for 20 min, red blood cells were lysed, trypan blue was added to quench extracellular fluorescence and samples fixed in 2% paraformaldehyde before acquisition on a BD Fortessa flow cytometer. Results are presented as the percentage of BCG-FITC+ events **(A)**, the percentage of BCG-FITC+ events associated with CD14+ cells **(B)**, and the percentage of BCG-FITC+ events associated with neutrophils [as defined by forward and side scatter among CD14 negative events **(C)**]. **(D,E)** Blood from 17 donors (4 independent experiments) was augmented with necrotic neutrophils, the supernatant of necrotic neutrophils, or medium alone, infected with 2 × 10^5^ CFU *M. bovis* BCG and incubated for 96 h at 37°C. Samples were centrifuged and supernatants stored at −80°C until analysis by ELISA for tumor necrosis factor (TNF) **(A)** or interleukin-10 **(B)**. Lines represent means, *p < 0.05 **p < 0.01, ***p < 0.001.

The addition of necrotic neutrophils reduced the overall percentage of events associating with BCG-FITC+ (mean ± SD percentage 4.51 ± 1.15 versus 6.82 ± 2.21 with no augmentation, *p* = 0.047, and 6.11 ± 0.88 with viable neutrophil augmentation, *p* = 0.027; Figure 5A). However, among BCG-FITC+ events, viable—but not necrotic—neutrophils reduced relative phagocytosis of BCG by CD14+ cells after 1 h of incubation (mean ± SD percentage of BCG-FITC+ events associating with CD14+ monocytes: in non-augmented blood 3.62 ± 0.98, in viable neutrophil-augmented blood 1.24 ± 0.38, *p* < 0.001 versus non-augmented, in necrotic neutrophil-augmented blood 2.89 ± 075, *p* < 0.01 versus viable-augmented; Figure 5B). Correspondingly, the percentage of BCG-FITC+ associating with neutrophils significantly increased with the addition of viable neutrophils (mean ± SD percentage: in non-augmented blood 69.72 ± 8.90, in viable neutrophil-augmented blood 82.95 ± 3.74, *p* < 0.001 versus non-augmented, in necrotic neutrophil-augmented blood 74.09 ± 7.72, *p* < 0.01 versus viable-augmented; Figure 5C).

### The Immunosuppressive Effect of Necrotic Neutrophils Requires Direct Contact With Cellular Debris

We hypothesized that some of the immunosuppressive effect of necrotic neutrophils might be mediated *via* a soluble factor, as has previously been described with other organisms (20). We therefore added necrotic neutrophils, their supernatant, or medium alone to aliquots of blood in 17 donors before incubation with *M. bovis* BCG for 96 h. Supernatants were then aspirated and assayed for TNF and IL-10 using ELISA.

As shown in Figures 5D,E, the addition of necrotic neutrophils to blood resulted in reduced TNF and increased IL-10 similar to previous results (mean ± SD TNF concentration in non-augmented blood 1,069 ± 1,351 pg/ml, in necrotic neutrophil-augmented blood 613 ± 814 pg/ml, *p* < 0.05; mean ± SD IL-10 concentration in non-augmented blood 899 ± 478 pg/ml, in necrotic neutrophil-augmented blood 1,236 ± 870 pg/ml, *p* < 0.05). However, addition of the supernatants of necrotic neutrophils alone did not have a significant impact on cytokine levels, albeit trends were evident (mean ± SD TNF concentration 713 ± 680 pg/ml, mean ± SD IL-10 concentration 1,040 ± 582 pg/ml).

## Discussion

Both viable (1) and necrotic (16) neutrophils are found in large numbers at the sites of human tuberculosis disease, where they will interact with mycobacteria and other cells of the immune system. We therefore investigated the impact of these cells on the control of mycobacteria and associated immune responses in a human blood model.

We have previously reported that neutrophils are crucial for the control of mycobacterial growth in human blood (21), and we here confirm and extend that finding *via* both depletion and augmentation experiments. Depletion of CD15+ granulocytes from blood has a greater adverse effect on mycobacterial control in whole blood than depletion of CD4+, CD8+, or CD14+ cells. It was interesting to note that depletion of CD15+ cells has relatively little effect on the cytokine and chemokine profile of *M. tuberculosis*-infected blood, suggesting that the mechanism of restriction of mycobacterial growth by neutrophils is likely to be directly mycobactericidal (or perhaps relates to cytokines and chemokines not measured here). Depletion of CD14+ cells and CD4+ cells did significantly impair the release of cytokines and chemokines, but this apparently had less effect on bacillary metabolism. These results raise the possibility that neutrophils, as the dominant cell type in human peripheral blood, are of importance for preventing hematogenous dissemination of bacilli. We note that, especially in areas of high endemicity, tuberculosis is noted not uncommonly as a cause of neutropenic sepsis (22, 23), while neutropenia is also associated with disseminated non-tuberculous mycobacterial infection and poor outcome from such infection (24).

Consistent with these findings, the addition of viable neutrophils to blood significantly improved control of mycobacteria. However, this intervention suppressed inflammatory responses and was associated with reduced phagocytosis of mycobacteria by CD14+ cells (organisms were instead taken up by neutrophils, which is likely to be simply a consequence of the relative numbers of each cell type). We propose that early intracellular killing by neutrophils results in less subsequent activation of innate and acquired immune responses. There may also be a contribution from the immunosuppressive effects of programmed apoptosis, although we have not specifically assessed this. It is interesting to note that *in vivo* data demonstrate poorer local IFN-γ response from CD4+ T cells in bronchoalveolar lavage samples from cavities, where neutrophils dominate (8). We note that the neutrophil augmentation experiments impacted on cytokine responses far more than neutrophil depletion experiments, which might reflect the greater absolute change in cell count of this manipulation.

We have observed (12) and others have also reported (13, 25) accelerated neutrophil necrosis in HIV-infected persons, and have hypothesized that this may be important in the aberrant host response to pathogens. Animal models also link neutrophil influx and associated necrosis to poor outcome (11), while neutrophilia in active tuberculosis correlates with poor outcome (4). We show here that the addition of necrotic neutrophils to blood, even in healthy donors, enhances the metabolism of *M. tuberculosis* while supressing protective cytokine responses believed to contribute to protection and increasing the release of immunosuppressive IL-10. We have previously demonstrated that the addition of necrotic neutrophils directly to mycobacteria does not restrict their growth by 1 or 24 h (2), and thus the effects seen here in the whole blood model are likely to be mediated *via* an impact on the responses of other immune cells to *M. tuberculosis* infection.

Simultaneously, growth factors (G-CSF and GM-CSF) and the chemokine CCL2 are also increased. Although favorable roles have been suggested for some of these molecules in mycobacterial infection, for example GM-CSF (26), their predominant role at sites of inflammation is to attract more cells—including more neutrophils.

The addition of necrotic neutrophils resulted in a global decrease in phagocytosis, with less cell events associating with organisms than with either viable neutrophil or no augmentation. However, there was not a specific effect on CD14+ cells. We therefore suggest that necrotic neutrophils reduce phagocytosis both by viable neutrophils (hence reducing early killing) and by mononuclear phagocytes (impairing protective cytokine responses), presumably because phagocytes internalize neutrophil debris instead. Indeed, the immunosuppressive process (reduction in TNF production and increase in IL-10), at least with BCG, required direct contact with cellular debris. We had hypothesized a directly suppressive role of neutrophil contents such as HNP 1–3, as previously described in other systems (20), but this does not appear to be a dominant mechanism in our BCG experiments (unfortunately we were unable to perform these same experiments with *M. tuberculosis*). Recent data have suggested an additional mechanism wherein necrotic neutrophils interfere with the phagosomal processing of *M. tuberculosis* by macrophages and thereby subsequent control of the mycobacteria (27).

We thus propose that in the context of active tuberculosis with necrotic neutrophilic inflammation, as may be seen severe tuberculosis or certain pathological states, there will be impaired mycobacterial control, reduction of protective but enhancement of immunosuppressive cytokine responses, and an ongoing influx of neutrophils to perpetuate this pathological cycle. This correlates with *post mortem* descriptions of tuberculous granulomas in HIV-1 co-infected patients which demonstrate extensive necrosis, marked neutrophil infiltration, and reduced TNF staining (13). Similarly, recent findings suggest that the CD15 neutrophil marker co-localizes with areas of necrosis and with IL-10 in tuberculous granulomas, and that the extent of this response correlates with *M. tuberculosis* burden (16). The results presented here suggest that the necrotic neutrophils may directly drive the IL-10 response and increased bacillary numbers rather than being simply epiphenomena.

This work has limitations, in addition to those already discussed above. Human hosts encounter *M. tuberculosis* at mucosal membranes rather than blood, although blood does represent a useful medium for study since it contains both innate and acquired immune system components. Heat shock is not the mechanism by which neutrophils die *in vivo*, but it does reliably induce necrosis within a short time: this method thus intended that autologous aliquots of viable neutrophils and whole blood should not experience significant cell death or other changes in the interim while necrotic cells were prepared. Depletion was not absolute for the target cells, but we felt that an approximately 90% reduction in cell count was likely to be biologically significant. As mentioned above, certain experiments were performed with *M. bovis* BCG rather than *M. tuberculosis* for reasons of biosecurity, and although we have previously seen similar results between these organisms in terms of neutrophil phagocytosis (2) and neutrophil control of mycobacterial luminescence (21), care should still be taken in extrapolating between organisms.

In summary, our data support the importance of neutrophils in the host response to tuberculosis both *via* a direct antimycobacterial activity and *via* an effect on the development of acquired immune responses. In particular, necrotic neutrophils impair mycobacterial control, reduce protective cytokine responses, and drive IL-10 and growth factor release: this pathological cycle may contribute to the negative association between neutrophilia and host prognosis (4).

## Ethics Statement

The studies were approved by the University of Cape Town Research Ethics Committee (HREC 545/2010) in South Africa and NHS Research Ethics Committee (REC 08-H0720-46) in the UK and performed in accordance with the Declaration of Helsinki. Participants provided written informed consent.

## Author Contributions

DL, JD, KW, RW, and AM conceived the experiments. DL, JD, and RG recruited participants and obtained samples. DL, JD, NB, and JN performed experiments. DL and JD performed data analysis. DL and AM wrote the draft manuscript and all authors contributed to manuscript revision, read and approved the submitted version.

## Conflict of Interest Statement

The authors declare that the research was conducted in the absence of any commercial or financial relationships that could be construed as a potential conflict of interest.
