# Supplementary material for: Differential Effect of Viable Versus Necrotic Neutrophils on Mycobacterium tuberculosis Growth and Cytokine Induction in Whole Blood
Source: Front Immunol. 2018 Apr 27;9:903. doi: 10.3389/fimmu.2018.00903 (PMC5934482; doi:10.3389/fimmu.2018.00903)
Supplement: Supplementary file 1 [file Image_1.PDF]

Supplementary Figure S1

A

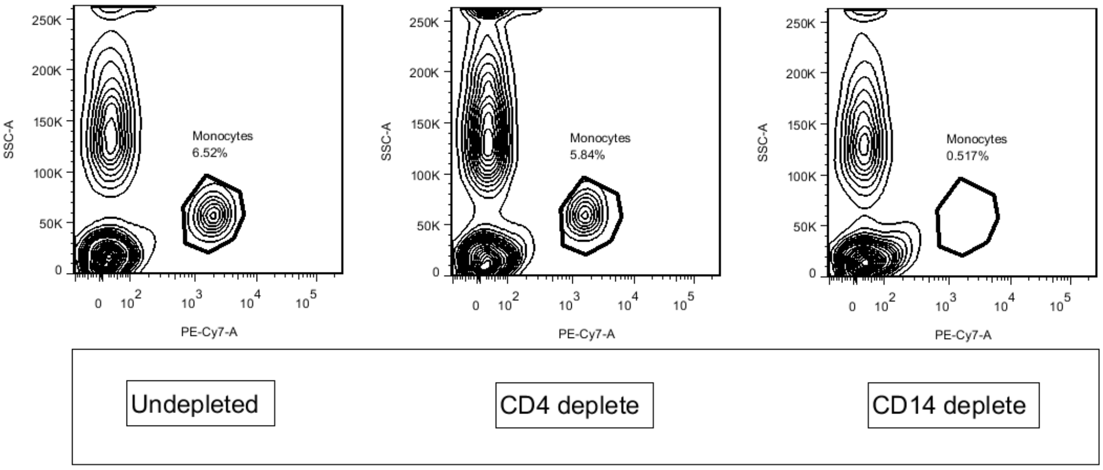

B

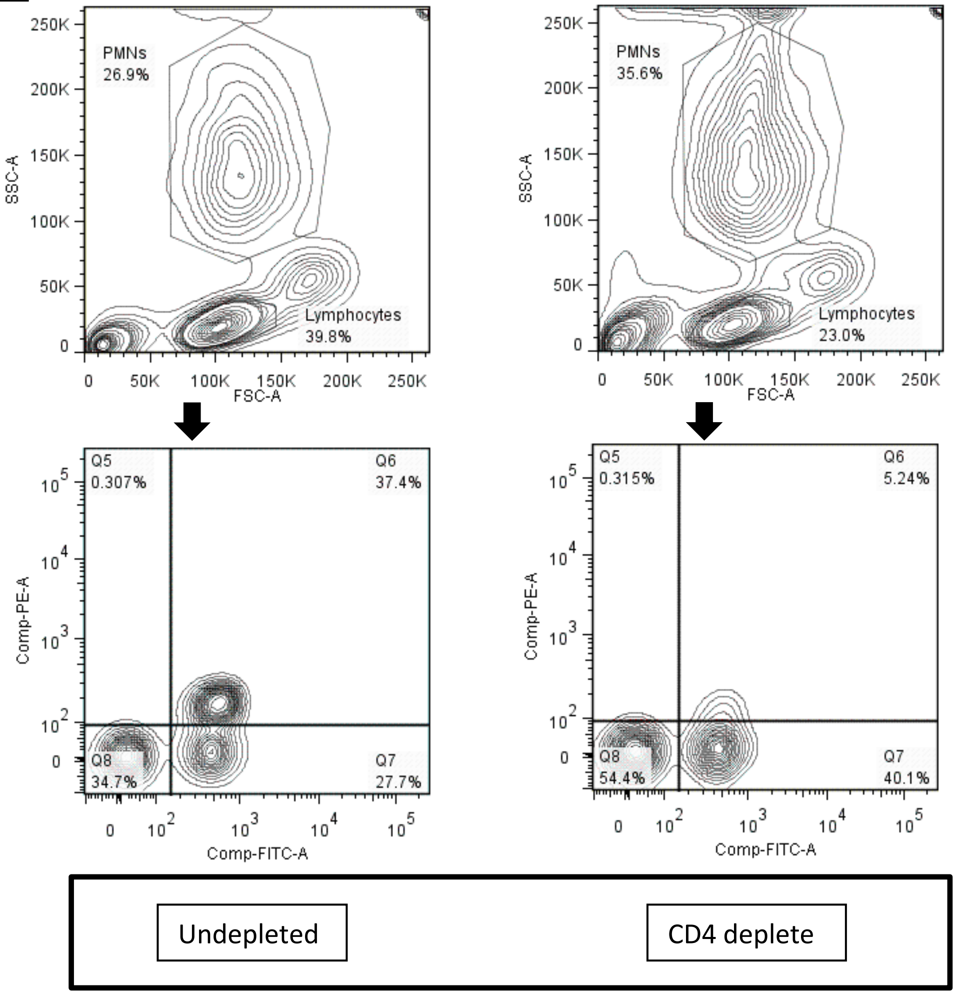

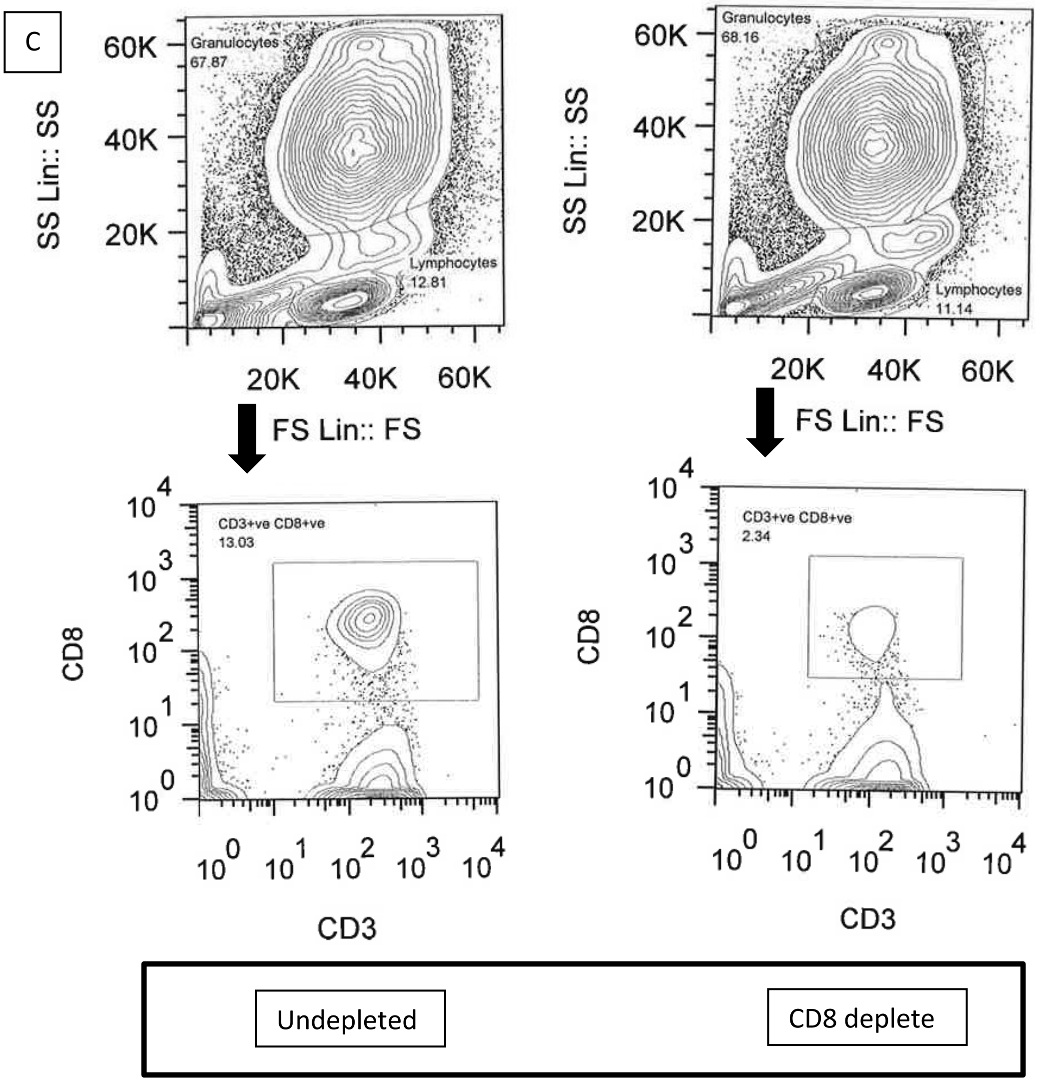

Flow cytometry on depleted whole blood. A. CD14 vs Side Scatter (SSC) defines monocytes. CD14 Depletion reduces population by 92%; no significant impact of CD4 depletion on monocyte population. B. Lymphocytes defined by Forward Scatter (FSC) and SSC are then gated on CD3 (FITC) and CD4 (PE) for undepleted and CD4-depleted blood. C. Lymphocytes defined by Forward Scatter (FSC) and SSC are then gated on CD3 and CD8 for undepleted and CD8-depleted blood.
