# Supplementary material for: Differential Effect of Viable Versus Necrotic Neutrophils on Mycobacterium tuberculosis Growth and Cytokine Induction in Whole Blood
Source: Front Immunol. 2018 Apr 27;9:903. doi: 10.3389/fimmu.2018.00903 (PMC5934482; doi:10.3389/fimmu.2018.00903)
Supplement: Supplementary file 2 [file Image_2.PDF]

Supplementary Figure S2

A

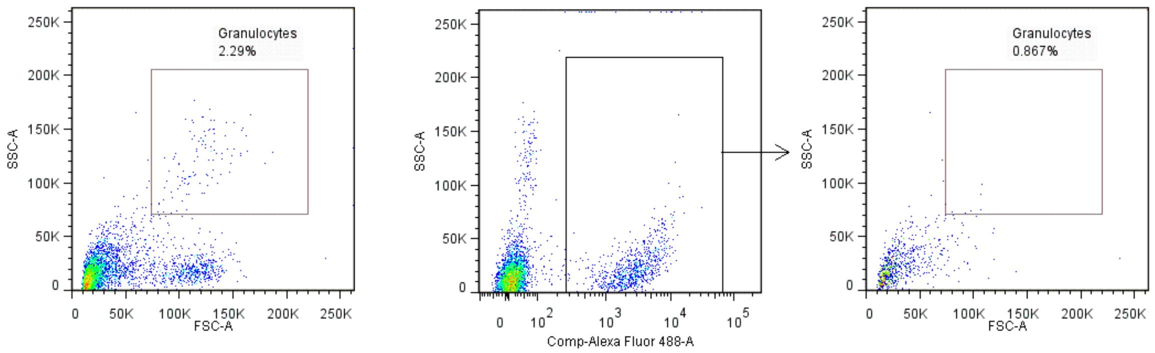

B

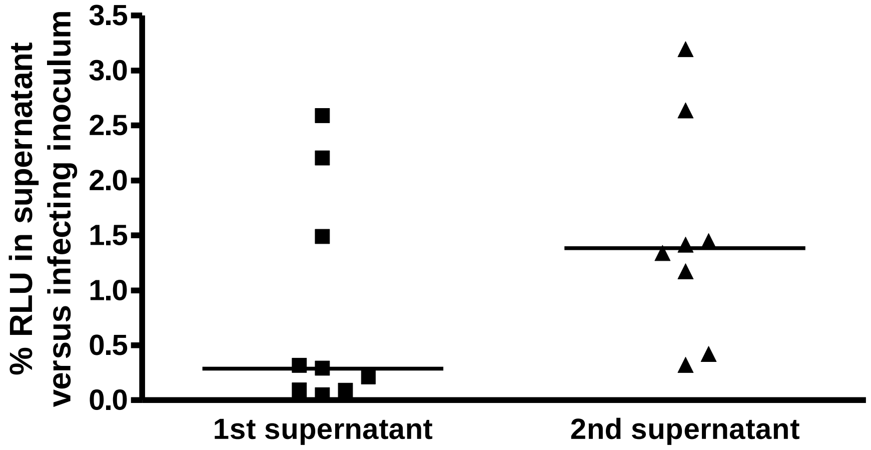

Despite effective cell lysis, mycobacteria are not significantly lost in supernatants. A. A sample of 450mcl blood + 450mcl RPMI was infected with BCG-GFP-Lux and lysis was undertaken with 10ml water before centrifugation and resuspension in 1ml PBS. Samples were acquired on a BD FACS Canto II flow cytometer. The panels demonstrate very few granulocytes as defined by forward and side scatter, and that <1% of GFP+ (Alexa Fluor-488+) events are potentially cell-associated. B. Samples of 450mcl blood + 450mcl RPMI were infected with BCG-Lux, centrifuged at 2000 x g for 10 minutes, supernatants removed (300mcl, 1st supernatant), lysed with 10ml water for 10 minutes and supernatants decanted (2nd supernatant). Luminescence was measured in duplicate on 100mcl of supernatant, mean results multiplied to the total volume of supernatant (i.e. x3 for 1st supernatant and x100 for 2nd Supernatant) and expressed as a percentage of the infecting inoculum. Lines represent medians.
