# Supplementary material for: Differential Effect of Viable Versus Necrotic Neutrophils on Mycobacterium tuberculosis Growth and Cytokine Induction in Whole Blood
Source: Front Immunol. 2018 Apr 27;9:903. doi: 10.3389/fimmu.2018.00903 (PMC5934482; doi:10.3389/fimmu.2018.00903)
Supplement: Supplementary file 3 [file Image_3.PDF]

Supplementary Figure S3

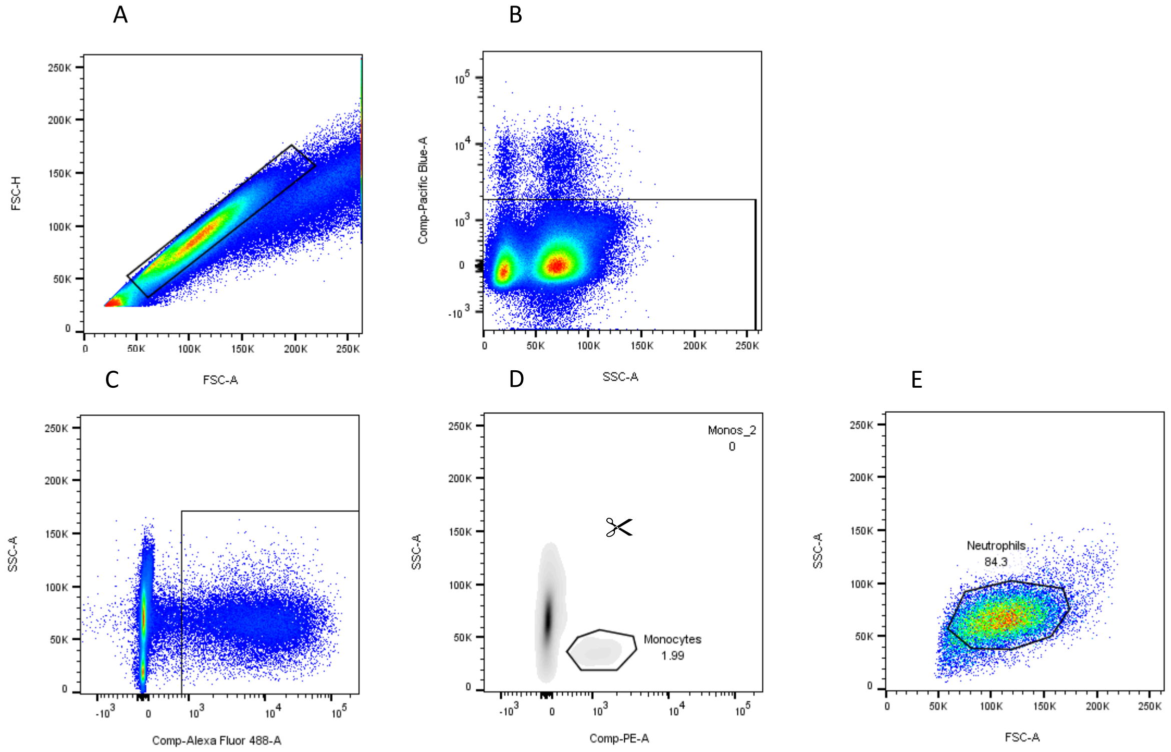

Gating strategy for phagocytosis assay. A. Singlet events are gated on the basis of Forward Scatter (FSC) Area (A) versus Height (H). B. Dead cells are excluded on the basis of positivity for Fixable Viability Stain 450 (Pacific Blue signal). C. Within live singlets, events associated with organisms are gated on the basis of FITC (Alexa Fluor 488) positivity. D. Within FITC+ events, cells are gated on the basis of positivity for CD14-PE. E. After exclusion of CD14+ events, neutrophils were gated via FSC and Side Scatter (SSC).
