# Supplementary material for: Differential Effect of Viable Versus Necrotic Neutrophils on Mycobacterium tuberculosis Growth and Cytokine Induction in Whole Blood
Source: Front Immunol. 2018 Apr 27;9:903. doi: 10.3389/fimmu.2018.00903 (PMC5934482; doi:10.3389/fimmu.2018.00903)
Supplement: Supplementary file 4 [file Table_1.docx]

**Supplementary Table S1**

p- and q-values for Principal component Analysis of raw Luminex™ data from augmentation experiments.

| **Analyte** | **p-value** | **q-value** |
| --- | --- | --- |
| CCL2 | 0.0061 | 0.0132 |
| CCL3 | 0.000769 | 0.00399 |
| CCL5 | 0.00103 | 0.00399 |
| CXCL10 | 0.00799 | 0.016 |
| Eotaxin | 0.000387 | 0.00251 |
| FGFb | 0.00222 | 0.00641 |
| G-CSF | 0.00451 | 0.0107 |
| GM-CSF | 0.00107 | 0.00399 |
| HGF | 7.029 x 10^-6^ | 0.000116 |
| IL-1β | 8.932 x 10^-6^ | 0.000116 |
| IL-10 | 0.00138 | 0.0045 |
| IL-12p40p70 | 0.00247 | 0.00641 |
| IL-2R | 0.000257 | 0.00223 |
| IL-7 | 0.0199 | 0.0345 |
| TNF | 0.00962 | 0.0179 |

CCL = C-C chemokine ligand; CXCL = C-X-C chemokine ligand; FGFb = fibroblast growth factor-basic; G-CSF = Granulocyte-colony stimulating factor; GM-CSF = Granulocyte Macrophage-Colony Stimulating Factor; HGF = hepatocyte growth factor; IL = Interleukin; IL-2R = Interleukin-2 Receptor; TNF = Tumour Necrosis Factor.
